# An Overview of Sensors, Design and Healthcare Challenges in Smart Homes: Future Design Questions

**DOI:** 10.3390/healthcare9101329

**Published:** 2021-10-05

**Authors:** Badziili Nthubu

**Affiliations:** Imagination Lancaster, Lancaster University, Bailrigg, Lancaster LA1 4YW, UK; b.nthubu@lancaster.ac.uk

**Keywords:** design, sensors, smart healthcare, data, visualizations, smart homes

## Abstract

The ageing population increases the demand for customized home care. As a result, sensing technologies are finding their way into the home environment. However, challenges associated with how users interact with sensors and data are not well-researched, particularly from a design perspective. This review explores the literature on important research projects around sensors, design and smart healthcare in smart homes, and highlights challenges for design research. A PRISMA protocol-based screening procedure is adopted to identify relevant articles (*n* = 180) on the subject of sensors, design and smart healthcare. The exploration and analysis of papers are performed using hierarchical charts, force-directed layouts and ‘bedraggled daisy’ Venn diagrams. The results show that much work has been carried out in developing sensors for smart home care. Less attention is focused on addressing challenges posed by sensors in homes, such as data accessibility, privacy, comfort, security and accuracy, and how design research might solve these challenges. This review raises key design research questions, particularly in working with sensors in smart home environments.

## 1. Introduction

With the ever-increasing population and old age [1], the healthcare system is overwhelmed with caring for the elderly and vulnerable groups in hospitals, nursing homes and clinics [2]. The UK National Health Service (NHS) spends double as much on old-age retired people than on the working-class population [3]. In the US and China, the population over 65 is estimated to double by 2040 [4]. The world’s old-age population suffers from age-related conditions such as diabetes, obesity, depression, dementia, hypertension, stroke and Parkinson’s disease [5,6,7,8,9,10], making healthcare costly. For example, falls were estimated to be costing the UK NHS close to £2 billion and the USA close to $34 billion per year [11,12]. As a result, researchers are exploring ways to reduce the movement between home and healthcare facilities [13,14] by focusing on providing smart healthcare in homes [15,16,17]. More recently, attention has been focused on exploring the provision of affordable and non-obtrusive ubiquitous sensing technologies in home environments [18].

###  Smart Home Healthcare

In [19], the term smart homes means “a special kind of home or residence equipped with sensors and actuators, integrated into the infrastructure of the residence, intended to monitor the context of the inhabitant to improve his or her experience at home”. Smart home healthcare is about using a network of intelligent devices, e.g., sensors and actuators, to enhance a home’s functionality, and improve the health and well-being of occupants and the efficiency of healthcare service delivery [20]. Previous work indicates that the increase in healthcare technologies facilitates self-monitoring, self-care, independent living and social connectedness in smart homes [13,21,22,23,24]. These healthcare technologies combine sensors, wireless communication and machine learning techniques [25]. Although there have been advances in smart home healthcare for the ageing population and people living with disabilities, these smart technologies still need to be user-oriented to properly manage home conditions [26]. Some studies show that younger people predominantly design sensor technologies, which leads to a digital divide between older people, i.e., the consumers of technology, and younger people, i.e., technology developers [27]. An in-depth survey paper about IoT for smart healthcare highlights that a solution is needed to reduce the healthcare system pressures whilst providing quality healthcare in smart homes [28]. Although sensing technologies promise a remarkable improvement in preventing chronic death and general ill-being, much work is now needed to engage patients in designing context-relevant IoT experiences [29,30].

This review paper analyzes the sensing, design and smart homes healthcare literature to assess existing research and challenges relating to IoT devices deployed in homes. As technologies advance, it is vital to engage users in smart homes’ design to create an engaging and contextually meaningful environment, hence the need to explore design research directions in this complex IoT captured healthcare environment. Recently, although designers in healthcare are gaining the necessary skills in co-design and user-centered design, they still cannot fully engage other stakeholders in the early stages of healthcare IoT projects [31] (p. 267). There is more room for design in this area, particularly in integrating multiple actors [32], exploring new ways to learn and working fluidly with others. It is important to consider using design methods in tackling the morass of IoT devices deployed in smart homes because design is useful for making sense of humans, systems and problems [33].

This review paper suffers from some limitations. First, the paper’s systematic literature review mainly focuses on two primary databases, i.e., ACM digital library and ScienceDirect. A few papers are also considered from databases such as GoogleScholar, etc., which are cited in the introduction and discussion sections. Second, we arranged our search based on four keywords, i.e., design, sensors, smart healthcare AND smart homes. The inclusion of other related keywords may have also resulted in relevant papers. Third, although the paper aimed to explore challenges in sensors, design and healthcare devices in smart homes, extending the search further to cover more ground on areas of smart home healthcare such as context and situational awareness might have improved the review. Fourth, the review only covers papers from 2015 to 2020, and hence some studies could have been overlooked. Future reviews may expand the search period to cover other publications which may be relevant to this topic.

In the next sections, the methods used to survey the literature are discussed, and then existing research and challenges in sensors, design and healthcare data between 2015 and 2020 are assessed. Finally, the review discusses the design research opportunities and highlights future research questions for designers interested in connected smart homes.

## 2. Materials and Methods

This review paper used a PRISMA (Preferred Reporting Items for Systemic Reviews and Meta-Analysis) protocol for screening relevant literature [34]. The review mostly limited the search to journals and conference papers from ACM digital library and Science Direct using the following keywords: “design, sensors, smart healthcare AND smart homes”. This paper selected 30 articles from each year beginning from 2015 to 2020.

**Figure 1:**
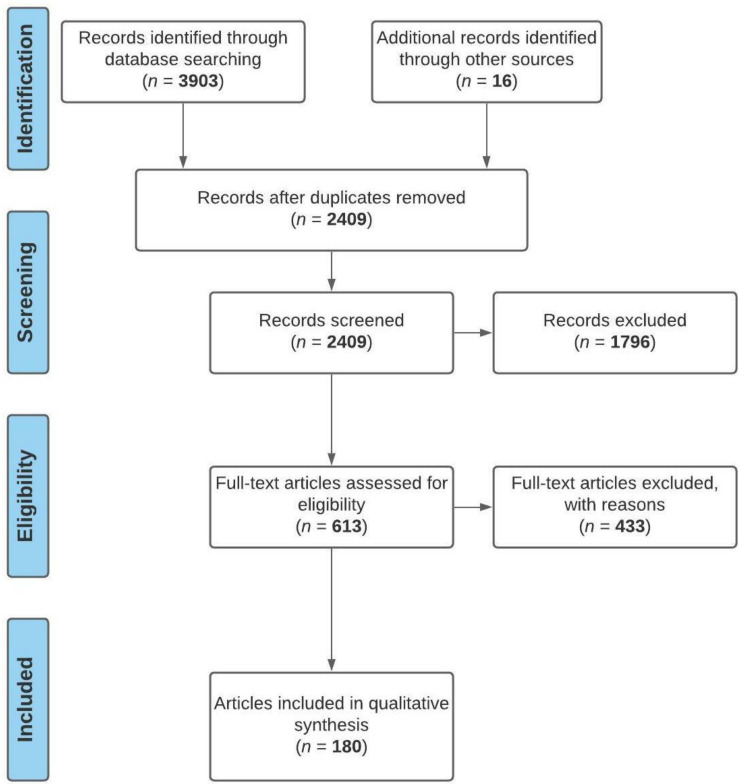
PRISMA protocol for screening relevant literature.

A total of 3919 articles were screened based on the keywords, abstracts and titles relevant to sensors, design and healthcare in smart homes (Figure 1). The screening process excluded 1796 papers that were irrelevant and duplicates. Further screening was performed using an abstract check for paper eligibility, and 433 papers were excluded. The exclusion covered articles that did not report important sensor projects deployed in homes and design and care data related to challenges associated with these IoT devices. Only 180 papers were included in the qualitative analysis because they met the set criteria.

After selecting the materials, i.e., review papers for analysis using the PRISMA protocol, the thematic method was used to explore data in NVivo 12 software. This method includes a detailed reading and coding (labeling relevant ideas related to the aim) of papers. The generated codes were further reduced into related clusters and re-labeled to represent a group of codes in a cluster. The clusters were assessed based on hierarchical charts (sunburst layout) for visual and pattern identification [35]. This was important to reduce the volumes of data and narrow the search for important ideas raised by other researchers.

The circular layout method [36] was deployed to visualize connections between important work carried out and the distribution of important authors in Gephi 0.9.2 software. This visualization method was essential to show hidden connections between authors and important projects. The circular layout also highlights significant connections related to the research aim. Circular layouts have been identified as crucial in exploring key insights in connected things [37,38]. After generating themes, the Venn diagram method, called the ‘bedraggled daisy’ explained by Luker [39] as a ‘Venn diagram on steroids’, was used to reveal the relationship between themes and their significance. Venn diagram themes help to put together ideas and identify relationships in a non-linear way, using two or more sets of Venn layouts. The ‘bedraggled daisy’ overlapping ovals help to organize themes and identify relationships between challenges in deploying sensors, design and healthcare data. Venn diagrams help to organize and visualize relationships between different insights [38].

## 3. Results

**Figure 2:**
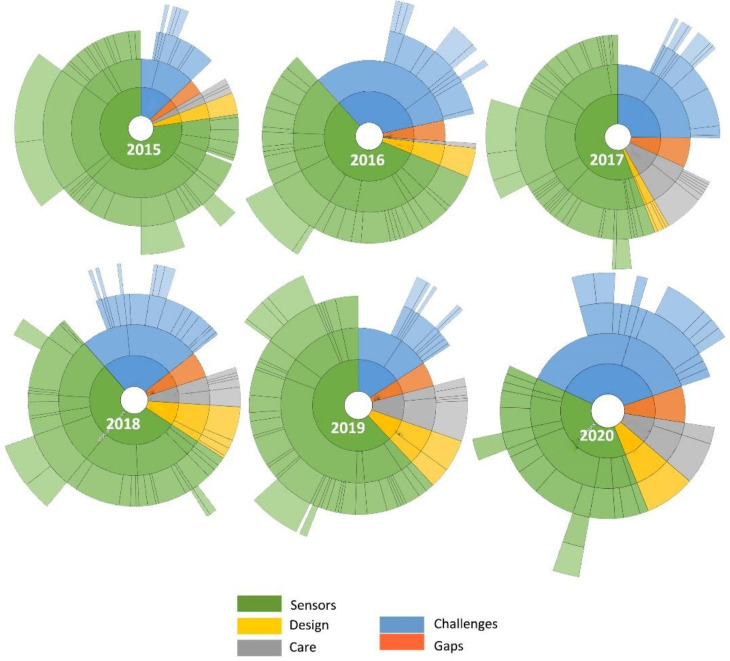
Visualization results on design, sensors and healthcare performed between 2015 and 2020.

Based on the analysis of review papers, Figure 2 shows a general overview of work carried out and challenges using hierarchical charts. These results cover the important work performed to develop ubiquitous computing sensors in a home environment from 2015 to 2020. It seems that much attention has been targeted at developing sensing technologies in ambient assisted living environments. In comparison, less attention was focused on user design approaches and healthcare data utilization in smart home environments between 2015 and 2017. There was a slight increase in design-led research between 2018 and 2020 papers. Additionally, less focus was placed on healthcare data utilization between 2015 and 2016 compared to 2017 and 2020. The latter years experienced much research, probably because of increased IoT care demand, prompting much research in design, data analytics and security. Figure 2 also highlights a recent increase in sensors, design and healthcare data challenges and gaps in 2020 compared to the previous years. These results imply that much work needs to be carried out in design research to address the IoT challenges and gaps.

**Figure 3:**
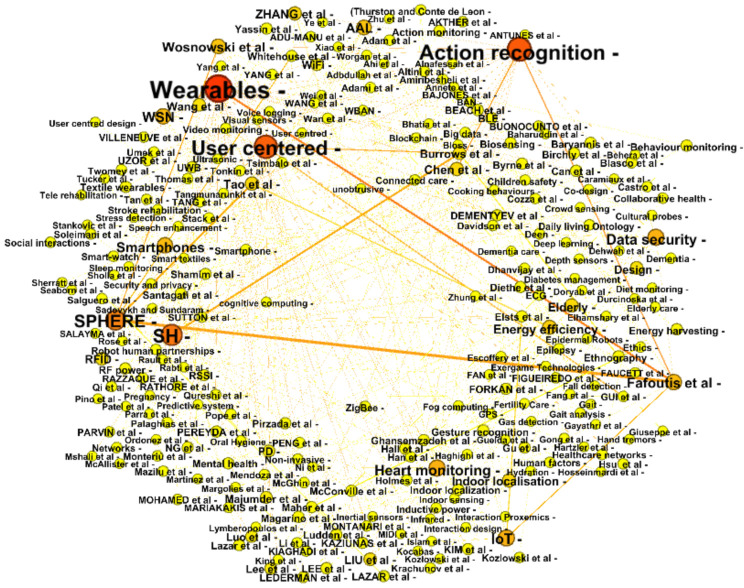
Visualization showing authors’ relationships with key projects.

Furthermore, the analysis identifies meaningful connections between IoT projects and key researchers using a force-directed layout (Figure 3). Based on these results, Fafoutis et al. [5,21] from the SPHERE project have a high degree, indicating greater influence in wearables and IoT research than other researchers. Still connected to the SPHERE project, Woznowski et al. [3] and Tonkin et al. [18] have a greater influence in action recognition research in smart homes. There is a significant link between the SPHERE project and design challenges. For example, they focused on the SPW-2 project to address design issues raised by users during the SPW-1 project. Other notable researchers such as Chen et al. [8] have a greater influence on action recognition research. Stack et al. [12] and Ni et al. [16] have a bigger influence on user-centered and ethnography research in smart homes. Regarding energy efficiency, Fafoutis et al. [21] and Tao et al. [22] have a more significant influence. Therefore, these results highlight that wearables, followed by action recognition and user-centered design, are the most researched areas. Furthermore, the results also reveal that the SPHERE project has published significant works in wearables, user-centered design and action recognition challenges. The circular layout method reveals important connections between projects and key researchers.

**Figure 4:**
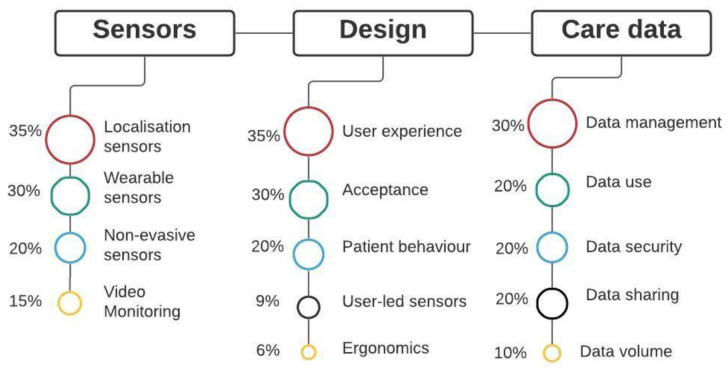
Results showing the main themes and sub-themes.

The thematic analysis identified subthemes related to sensors, design and care data themes (Figure 4). Under the sensor theme, 35% of studied papers highlighted challenges associated with the localization sensors, 30% with wearable sensors, 20% with non-evasive sensors and 15% with video monitoring. Under the design theme, 35% of the articles highlighted challenges with user experience, 30% with acceptance, 20% with patient behaviours, 9% with user-led sensing and 6% with ergonomic challenges regarding sensor design. Under the healthcare theme, 30% of the articles highlighted data management challenges, 20% on data use, 20% on data security, 20% on data sharing and 10% on data volume. Based on these results, it is clear that the localization and wearable sensors’ challenges are the most researched under IoT devices. Whereas under the design theme, user experience and acceptance are the key challenges identified. In healthcare data, data management and use are the main challenges mentioned by most researchers. These results provide an overview of what themes are important in IoT research in smart homes. Next, the paper discusses subthemes in detail.

### 3.1. Sensors in Smart Homes

Sensors are classified into location, physiological, image, inertial, environmental, binary and tags. Indoor location sensors include wearable and ambient sensors [40]. Localization methods use Bluetooth, radio frequency identification (RFID), passive infrared, Zigbee, Wi-Fi, ultra-wideband (UWB), frequency modulation, inertial sensors and cameras. Wireless and inertial sensors are the most popular methods [41]. The use of Wi-Fi signals as a localization method has been widely investigated [42,43,44,45,46]. However, indoor localization remains a less developed area in healthcare sensing than the outdoor experience achieved through the global positioning system [47]. Since GPS cannot be used indoors, solutions such as LoCATE have been proposed to track patients using Wi-Fi [48,49]. Indoor positioning methods such as accelerometers and gyroscopes have also been deployed [50]. These are used in homes to monitor daily activities, i.e., walking, sleeping, eating, climbing, sitting, cooking and standing [17]. Akther et al. [17] proposed the mORAL model for inferring oral hygiene behaviors. The SPHERE project deployed several wearable sensors which collected inertial data, environmental data and visual data to monitor the behavior of users [19].

Other localization systems include ultra-wideband. This was deployed in a project called SUGAR as an indoor navigation system for visually impaired people [51]. Previous studies report wearables such as RF-Kinect to capture human body movements [52], RF-focus for baggage sorting [25], Tagyro to remotely capture the orientation of objects and TagFree, which is a non-wearable RFID tag [53]. Researchers report that ZigBee is amongst the most used small, low-cost, low-power, short-range wireless technologies [54,55]. Infrared signals and images are also used to localize and track humans indoors [56,57,58].

Gualda et al. [59] presented a locally referenced ultrasonic system for localization and navigation. For example, the U-wear network platform is used as a more secure communication system than radiofrequency [60]. Bluetooth low energy is used to obtain the coordinates of object locations using a BLE beacon inside the building [61,62]. For example, Apple’s iBeacons provide location-based information and services to iPhones and iOS devices [63].

Physiological sensors measure respiratory and cardiac rhythm to assess sleep patterns [64], stress management and predict respiratory-related diseases [65]. There are currently many wearable and non-wearable devices for sensing respiratory and cardiac rhythm on the market. Commercial physiological monitoring systems are mostly in activity recognition, vital sign monitoring, fitness and blood pressure monitoring [16]. Smartphone sensors are primarily used for monitoring these physiological signals. Lee et al. [24] designed a low-cost, adaptable and personalized remote monitoring system to be deployed in remote areas through mobile devices.

Wearables have various healthcare sensors to measure and monitor heart rate, blood pressure, temperature and glucose levels [66]. HeartSense was deployed in 836 experiments with different contexts to perform the heart rate estimations using smartphones and gyroscope sensors [67]. Furthermore, researchers developed the epidermal robot at the MIT media lab to measure body parameters [68]. Verity is an ambient assisted living platform deploying wearable sensors with accelerometers and piezo-resistive sensors for detecting falls and heart rate [3,5,19].

Non-wearable sensors are non-invasive due to minimal contact with users. Microsoft Kinect has been widely adopted as a low-cost, non-wearable device that monitors users’ adherence to drugs [69] and monitors older peoples’ meal intake [70]. A textile-based sensor system was also proposed to measure the physiological signals as a non-invasive and low-cost solution [10]. Yang et al. [71] developed mmVital, a non-invasive vital sign monitoring process to monitor sleep. Other non-invasive technologies include TagSleep, which uses RFID tags placed near the users [72]. Non-invasive sensing is also achieved through smartphone sensors [64,73,74]. Miniature snap-action switches were also deployed in a SPHERE project for activity recognition [75].

Besides the sensor deployment, the survey also considered IoT devices’ context and situational awareness as important aspects of smart home healthcare. This is because the success of smart home healthcare depends on the effective awareness of the environment [13]. The awareness of the environment entails good knowledge about the user and their daily behavior based on perceptions, context, environment and prior knowledge [13]. Therefore, context-aware frameworks can automate the home environment, monitor users’ health and predict possible risky behaviors [76]. For example, these frameworks are important for locating and monitoring dispositions of monitored individuals. Previous literature highlights different ontologies and context-aware middleware used for context and situational awareness in homecare applications. According to Forkan et al. [77], a cloud-oriented context middleware was proposed for assisted living to learn normal behaviours and identify abnormal patterns in IoT data to inform other decisions and actions. Pung et al. [78] also proposed a context-aware middleware for elderly homecare to provide data acquisition, storage and reasoning services. Data reasoning is deducing relevant information from various data sources [79].

Nugroho et al. [80] used an ontology-based context-aware model for personalization of the sub-systems’ learning process and as a decision support tool for doctors and caregivers. Other ontologies used for reflective middleware applications include CAMeOnto, which is based on the 5Ws: who, when, what, where and why, and six contexts, i.e., user, activity, time, device, services and location [81]. De la Iglesia et al. [82] also used a content-aware system model, with non-intrusive devices based on the Big Data Context-Aware Monitoring (BDCaM) framework to monitor the neonate and air quality in their environment. In another study, MSSN-Onto is discussed as an example of an ontology-based approach for event processing in complex multimedia sensor networks [83].

In the following section, the challenges of sensors and context-aware systems in smart homes are discussed.

#### Sensor Challenges

**Figure 5:**
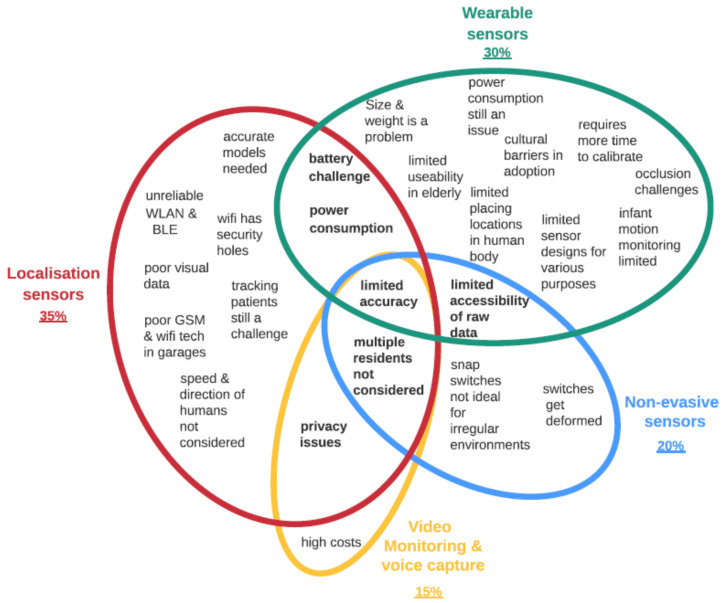
Highlighting sensor challenges in smart homes.

By using the Venn diagram, the analysis reveals the main challenge of limited accuracy in various sensors (Figure 5). Woznowski et al. [84] highlight that voice-enabled technologies do not always capture speech accurately because of different accents. Additionally, inaccuracies still exist in human activity recognition systems [14,41]. Wearables face occlusion challenges, especially devices worn on hands and legs, affecting the accuracy of data captured and persons’ daily activities [85]. Measuring the speed and direction of human movement in homes is still tricky [56], particularly detecting multiple residents’ patterns [86]. Using localization, video monitoring and non-evasive sensors is also a challenge because different people behave differently [63], e.g., people with Alzheimer’s and dementia [1]. Lack of elaborative contextual information in sharing data amongst multiple users (patients) was also cited as a challenge [87].

The visualization also shows that battery and power consumption challenges are common amongst localization (Wi-Fi detection methods) and wearable sensors [1,19,36,88]. Researchers attempted to develop wearable devices with ultra-low energy consumption using painted electrodes [89,90]. Furthermore, privacy concerns are alarming in indoor positioning systems, e.g., video monitoring sensors [84,91]. Deploying monitoring and voice-enabled technologies in homes remain intrusive and expensive [92]. Patients’ limited accessibility of raw data is a challenge in wearables and non-evasive sensors, especially in older people [19].

Regarding available context-aware systems, privacy and security of personal data are still challenging in using middleware in location and monitoring (Figure 5). Although middleware reduces the need for human involvement, more human interventions are required to ensure high levels of context awareness [93]. Sykes et al. [94] also highlight that using Bluetooth low-energy iBeacons to provide a contextual and personalized experience for users, e.g., tracking patients, is possible in open spaces, but it can be a challenge in small rooms due to high interference.

Other challenges highlighted in each theme are as follows (Figure 5): localization sensors face challenges when WLAN and BLE are unavailable [50], and Wi-Fi still poses security holes [49]. Visual data from cameras and measurements of humans’ speed and direction are still lacking [56] due to the difficulty in tracking multiple residents in smart homes [1].

Common wearables’ challenges include units’ size and weight, which causes discomfort in use [88,95], thus limiting useability in elderly persons and infant motion monitoring [8,19,58]. Other challenges include limited placing locations on a human body, limited designs for various purposes [14] and calibration challenges [14,85]. It was recently reported that stretchability, low-cost materials, ultrathin materials, biodegradable and biocompatible and self-healing materials are important in sensor design to improve comfort and use [63,96]. Although non-evasive sensors have minimal contact with users, there are still challenges with collecting data using switches. Cupboard snap-action switches are not ideal for unstable kitchen environments (Figure 5). Whitehouse et al. [75] report that the switches either become damaged or produce noisy observations. All of the above challenges require an effective design approach for sensor design and deployment.

### 3.2. Design

The widespread use of wearable technology in healthcare makes it an interesting design challenge. What care means for people affected, i.e., elderly, vulnerable groups (dementia, epilepsy) and caregivers, needs to be examined to shape smart home technologies [97]. Although technologies bring progress in healthcare, they also reduce the physicality of care, i.e., humans are physical and mostly relate well with physical things [98]. Increased use of technologies in homes may increase users’ risk of isolation [99]. Researchers are starting to focus on promoting human-to-human interactions using non-verbal body language with inertial sensors. Handling and understanding healthcare data requires professional medical knowledge, and patients seem to lack the motivation and tools to explore these data [87,100]. There is a need for mechanisms that allow people to interact with data instantly [101]. Mobile healthcare social network (MHSN) is a tool developed to promote sharing of experiences amongst users (elderly), and MOSKUS (Mobile Musculoskeletal User Self-management) is also cited as a solution to enhance the self-management of arthritis patients [40]. Some followed a co-design approach to develop a smartphone app for people with Down syndrome, to help them with nutrition decision-making [102]. Recently, co-design approaches were suggested as an effective way to engage users in data applications [103].

A literature survey on older adults shows distinctively different attitudes towards surveillance and on-body sensors [104]. These body sensors require the users to always carry or wear them, and older adults generally dislike wearing devices [23]. As a solution to provide a socially assistive service, the Hobbit robotic platform is one of the recent attempts to design human–robot interactions [105]. Some designed and implemented a platform to support the elderly in a smart home [106], but the system only catered for a single occupant, which may vary in functionality with multiple elders. As discussed earlier under sensors, context awareness seems to play a key role in healthcare monitoring in a home environment [13,84,107,108].

According to Ni et al. [109] (p. 2), context is “any information that can be used to characterize the situation of an entity”. Context awareness and interpretation are important in understanding complex situations for health assessment, e.g., night activities [110]. Nava-Muñoz and Morán [111] proposed CANoE, a model for Context-Aware Notification for Critical Environments. This model considers the context in delivery, content and presenting a notification based on the environment, elders and caregivers. Perceiving surrounding situations and activities is important to promptly respond to accidents and other adverse situations [112]. Ni et al. [109] proposed a three-layered context-aware system architecture to process data for context information, whereas Alirezaie et al. [113] presented a system called E-care@home, which is a context awareness IoT setting shifting from network-centric context awareness to data-centric context awareness. However, most of these context-aware systems still lack accuracy in interpreting a home environment’s complex contextual and situational conditions [110].

Researchers emphasize the need to focus more on end-user practices out of which healthcare data is constructed [114]. PRESENCE is one example of a personalized solution designed using a user-centered design approach [115]. Some authors also used a user-centered design approach to explore the self-annotation of smart home data [18]. This idea helps to explore the use of data. Noncontact ECG monitoring systems have also been developed through users’ responses to reduce contact with the skin [116,117,118].

Furthermore, a user-centered approach is suggested to provide people with dementia with social engagements [7]. The SPHERE project conducted user-centered design work to explore users’ experiences with technology [119]. In a different context, the RAS robot is a cyber-physical approach that offers support by sensing human behavior in adults and young people [120]. Nevertheless, in home-based rehabilitation technologies, factors such as social context and life experiences are less explored [11,121]. Kim et al. [122] investigated the design requirements for a self-monitoring system based on case studies with stroke survivors. Uzor and Baillie [11] also demonstrated using exergames to make users more involved with rehabilitation technologies. The SPHERE project emphasized a design-focused contextual understanding of community healthcare systems [30,101].

Ethics in healthcare data is increasingly becoming an issue of serious scrutiny [123]. Overcoming ethical barriers is challenging and may require a user- and context-sensitive trustworthy information system in homes [124]. Critical reflection is key to ethical design because it leads to conscious awareness and decision-making [125]. A recent study looked at ethics codes that address social concerns about technologies in communities [126]. Collaboration in healthcare policy reforms is suggested to develop an inclusive mobile healthcare system [122,127]. This may promote interactions between patients, clinicians, government policymakers, engineers and the wireless industry.

#### Design Challenges

**Figure 6:**
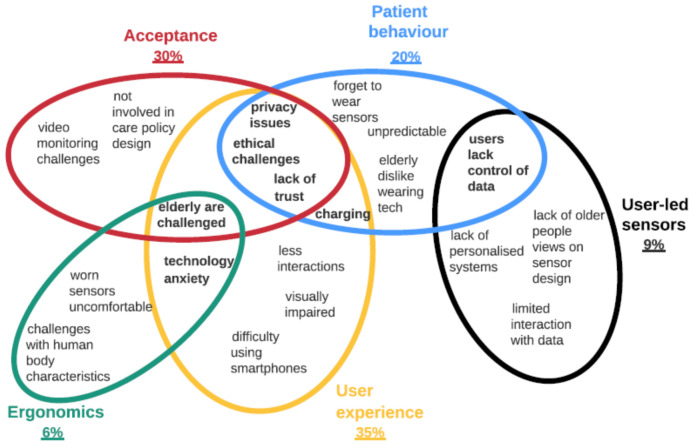
Highlighting design challenges in smart homes.

Five main themes emerge as design challenges in deploying smart home sensors, as highlighted in the Venn diagram (Figure 6). Privacy issues, ethical challenges and lack of trust are the most common among the three themes, i.e., patient behavior, acceptance and use (Figure 6). Patients’ privacy using vision sensors [91] and wireless technology [60] in homes is a design challenge because it affects patients’ trust and behavior in using IoT devices. Durcinoska et al. [128] highlight a challenge in measuring trust in healthcare networks. Ethical challenges also arise due to decision-making autonomy in IoT devices’ design and data ownership [129]. For example, the RAS robot may be deceptive, especially to naïve and vulnerable users of emotional attachment [130]. Lack of data control and uncertainty about how patient data is used affects their behavior and the acceptance of IoT devices [101,131]. Cozza et al. [125] also show that elders are considered people in need of technology solutions rather than empowering them with their data to solve problems independently.

Using the Venn diagram also highlights the main challenges common to at least two themes. Charging devices seems to be a major challenge related to patients’ behaviors and device use (Figure 6). This is because most wearable devices require constant charging, so older adults and people with disabilities, e.g., dementia, are more likely to forget to charge [83]. The elderly face challenges in operating wearable devices, e.g., smartphones and watches [14], which is associated with poor ergonomic features. Users perform the same behaviors differently depending on their physiological characteristics, contextual and situational conditions [63]. Context distribution through dissemination mechanisms such as visualizations can be complex to execute. Hence, Sykes et al. [94] suggest that different visualizations should be adopted in a more human, interactive and instinctive manner. Additionally, there is a need to develop care technologies that are simple, reliable and customized to individual needs [104]. Elderly patients and users are uncomfortable with intrusive technologies, such as tightly worn sensors [14,23].

Advanced sensing technologies lead to technology anxiety in elderly users [132]. The elderly view independence and autonomy as key, so technologies that promote these views will likely be accepted [124], but attitudes such as technology anxiety, beliefs and efficacy challenge their adoption rate [132]. Other challenges observed include users’ limited interactions with data, which may inform the development of useful sensors in homes (Figure 6). Solutions for using smart devices to enhance users’ interactions and data utilization efficiency remain limited [133,134]. Real-time behavior analysis in the elderly looking at their context specificities has also been reported as a challenge [135].

### 3.3. Smart Healthcare Data

Smart home healthcare extends the traditional hospital to a home, thus benefiting both the patient and the caregiver [136]. A smart healthcare system consists of different sensors, servers and the network to handle different data types [137]. These smart systems provide contextual information that can be processed and stored in cloud-based systems to provide real-time data for remote monitoring and care [138]. Baker et al. [28] discuss cloud-based challenges, such as the need for increased computational speed, decreasing computational requirements and high-level machine learning abilities. The authors further argue that cloud-based healthcare still needs a suitable encryption scheme relying on low-power sensors and cloud storage.

Recently, smart healthcare is shifting from cloud to fog computing [139]. According to Al Muhtadi et al. [140], fog computing is required because the cloud alone fails to satisfy users’ mobility support, location identification and low latency. Fog computing is an intermediate layer between the cloud and edge devices to reduce the amount of data transmitted to the cloud by processing part of it at the edge [139]. Tang et al. [141] investigated and implemented a fog-enabled smart health system for data sharing. Al Hamid et al. [142] proposed a tri-party agreement protocol based on the bilinear pairing cryptography. However, Rahimi et al. [143] discuss the challenges of security and privacy in fog computing, some of which are inherited from cloud computing. Al Muhtadi et al. [140] highlight that authentication and trust are vital challenges of fog computing and propose a subjective trust model to improve IoT security at the edge.

Smart home data are divided into active and passive. Active data involves patients, and passive data does not [144], which is a huge ethical concern in preserving patients’ privacy [103]. Various types of healthcare data can be collected using different sensors. Physiological data are mostly about heart rate, breath rate, body temperature and blood pressure. Physiological data are mainly captured through smart body wearables and biosensors [145]. Behavioral data are more concerned with the stress levels of a person. Environmental data are to do with indoor temperature, humidity and oxygen. Dietary data are a key area of home care, where sensors monitor nutritional intake [70,75] and analyze cooking behaviors for health monitoring [146]. Security is a major issue in the IoT data [10], particularly the control of access to healthcare data [98], which is highly private and sensitive [55]. Even though Fang et al. [147] propose an efficient scheme to provide fine-grained access control to their data stored on the cloud, Alami et al. [136] indicate that vulnerabilities in using symmetric cryptography where attackers can easily generate all the keys to break the privacy of the network are yet to be resolved. Using Radio Frequency (RF) technology in wearable devices is still vulnerable to eavesdropping by malicious agents [13,60]. Though multimedia devices provide rich data, they also suffer from privacy issues. To enable data sharing from private users, PoolView hides personal data while allowing community data sharing [148]. Blockchain is also suggested as a secure way to store healthcare data using distributed ledger and authentication systems [66,149,150,151]. Some authors proposed intelligent collaborative security models to minimize the risk [152]. However, Aceto et al. [103] recently reported that further work is needed to develop IoT security solutions because the current solutions are not scalable to the ever-increasing Big Data. IoT architecture remains highly hackable, and hackers can misuse patient information [108]. Liu et al. [19] reported that some wearable sensors’ personal data could leak through wireless channels to eavesdroppers even when the data is encrypted.

#### Smart Healthcare Data Challenges

**Figure 7:**
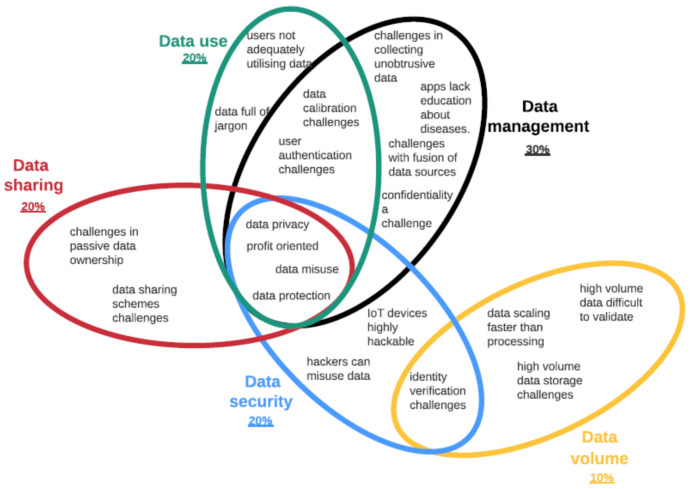
Highlighting healthcare data challenges in smart homes.

Four main subthemes (data privacy, profit orientation in user data, misuse and protection) are common to data management, data use, data security and data sharing themes (Figure 7). The Venn diagram reveals data management as the most discussed theme. Identity verification is common to data security and volumes [150]. The higher the volumes of data, the more challenging to secure [63]. Regarding data management, current work shows that there is still a challenge in using healthcare data to detect early health conditions in homes [153]. IoT devices, e.g., apps, lack feedback education systems to help users manage health conditions, e.g., early detection of epilepsy seizures [154]. As part of data management, care data calibration is also neglected [155], affecting decision-making and personalized care. The difficulty in collecting unobtrusive data in assessing behavior and physical situations in adults and people with disabilities is also problematic [23].

Observing the Venn diagram (Figure 7), data calibration and authentication challenges affect data management and use due to increased hackers and eavesdroppers [66,150]. However, jargon in healthcare data [100] and users not adequately utilizing data are huge challenges [87]. Challenges in passive data ownership [123,144] and sharing schemes [134] also affect data sharing amongst users. IoT devices are highly hackable even when applying blockchain technologies, and hackers can misuse healthcare records [66,123]. For example, hackers can use the care data to create fake IDs to buy drugs or access key assets such as banks [108]. Therefore, preserving data security and privacy is a big challenge [144]. The smart home area network integrates diverse and intelligent devices, services and technologies, hence the need to introduce external actors to manage the system [156]. Data volume is scaling faster than the devices’ processing capability [150,157]. This causes high-volume data storage challenges and difficulty in validating healthcare data [63].

## 4. Discussion

Creativity and innovation require new ways of working [158], and design offers the means to leverage new ways of working through expanded realms of action [159]. Although designers in healthcare have skills in user-centered design and collaborative design, there is insufficient action and involvement of all stakeholders in IoT design and adoption in smart home healthcare [31]. This is because involving end-users in healthcare systems is challenging [160]. Healthcare systems are traditionally built on notions of hierarchy and control [161], whereas IoT users in homes want autonomy and independence. Frailsafe is an example of a project in healthcare that intended to flatten hierarchies and reduce authority amongst team members, albeit engaging management was still a challenge [162]. Therefore, it is important to adapt to person-centric healthcare perspectives, e.g., decision-making with less asymmetrical power relations [163]. Evidence from this review shows that IoT devices and design research in homes can enhance assistive services to older adults and vulnerable groups to collaborative design activities. This will require better user-centered design tools to enhance understanding of human behavior, which is unpredictable and complex. Interpreting complex contextual and situational conditions of a home environment remains a challenge [112]. This challenge is partly because the available malwares have limited degrees of context and situational awareness [93]. Design research offers the opportunity to build connections and capacity in complex, transdisciplinary contexts [37,160].

Emotional links that users, i.e., elderly and vulnerable groups in smart homes, develop with assistive technologies inspire positive lifestyle and personal choices [164]. From this review article, it was found that sensors’ design and power consumption challenges affect users’ personal choice to use wearable devices. This is because wearable technologies may negatively impact users’ comfort and adoption patterns, thus raising future design questions: (1) How might design enhance the adoption, comfort and data accessibility in smart home environments? (2) Is there an innovative way elderly persons, caregivers and other smart home occupants’ views might influence the design of sensors deployed in homes? The Life 2.0 project attempted to offer innovative and user-friendly tools to create social interactions between ageing communities [165]. Further research is needed in engaging home users through simple design interaction tools, e.g., visualization techniques.

The review paper discussed a few non-obtrusive solutions to reduce discomfort posed by wearables, albeit these technologies also have privacy and security concerns. Many researchers indicate that monitoring patients remotely in their comfort is crucial. Some survey papers highlight the use of non-invasive methods such as depth approaches and snap switches to minimize the obtrusiveness of sensors in homes, but these methods also suffer from a lack of accuracy. Using videos to monitor patients in homes is also facing acceptance issues, especially in older persons. According to Sarantou and Pan [165], the elderly worked with designers to translate their experiences into service design and delivery, thus co-designing with all stakeholders affected. Another issue is that voice-enabled technologies do not always capture voices accurately because of different accents in homes. Therefore, future design questions will be around the following: (1) How might indoor monitoring of patients be carried out without intruding on their privacy and compromising their data? (2) Is there a way design approaches can enhance video acceptance in homes as a monitoring device? (3) How might the voice technologies be improved to accurately capture speech in homes with more than one occupant? The answers to these questions lie in how elderly and vulnerable groups in homes can act as social designers to address the needs they want answered for their futures [165]. Tailored needs and interventions focusing on individual experiences can be promoted through co-design and participatory activities [166].

Lack of patients’ data control and interpretation also contributes to a lack of trust in how data is used. This is because there are no effective interactive feedback tools to interpret data back to patients to instantly improve their health, e.g., healthcare data is full of jargon, which might contribute to low IoT adoption, particularly in older adults. Future design research may focus on the following questions to address these challenges: (1) How might design and visualization methods support healthcare networks to adapt to user requirements and needs? (2) How might visualizations be leveraged to enhance the healthcare ecosystem of patients, caregivers, family members, the wireless industry and healthcare practitioners? (3) How might design be leveraged to develop better devices for monitoring and communicating care solutions to users? Evidence from the review shows that challenges of visual thinking in healthcare could be addressed through refining the design thinking tools used [167]. Additionally, with the recent advances in fog computing, that is useful for short-term analytics at the edge of the cloud computing system, how data is communicated from this layer to users needs to be enhanced.

Handling healthcare data seems to be a major issue from the point of view of ethics of collecting, storing, securing, using and sharing data. Zanutto [168] argues that innovation needs to focus on the patients’ ability to translate care data into care practices, thus reconfiguring their relations with healthcare actors. There is still confusion on the ownership of passive data between patients, researchers and healthcare practitioners. Therefore, future research is now needed in using design methods to answer the following questions: (1) How might ethics be designed in the collection, storage and security of healthcare data in homes? (2) How can healthcare data be used and shared amongst the care networks safely and ethically? Exploring these design questions will significantly influence the development of a user-driven smart home environment instead of a technology-driven approach. Additionally, user-centered solutions may further enhance the fog-enabled healthcare smart home system for safe and secure data storage, processing and sharing. This is because cloud computing alone fails to satisfy users’ requirements [140]. Data visualization techniques can enhance questions on how healthcare data can be used and shared amongst the healthcare network. Data visualization can enhance the understanding of large-scale IoT data or Big Data from smart homes [169]. Better visualization techniques to process and communicate data at the edge have the potential to significantly satisfy user requirements and needs.

## 5. Conclusions

The major contribution of this review is that it provides insights to the readers about challenges in sensors, design and smart healthcare devices in smart homes. The paper also used a combination of the PRISMA protocol for screening relevant literature and visualization techniques to identify challenges of sensors, design and smart healthcare in homes. Although it is not an exhaustive survey, it provides an overview of the existing research about IoT devices and significant projects deployed in smart homes. It highlights the challenges of sensors, design and smart healthcare in homes and future research questions for design researchers. This review paper positions the role of design in smart homes as crucial towards making smart home constituents comfortable and safe around IoT devices such as sensors and actuators. This might be particularly significant to enable smooth collaboration between patients and their stakeholders. More attention is now needed in exploring data with users to address these emerging design research questions.

## References

[1] Ghamari M., Janko B., Sherratt R., Harwin W., Piechockic R., Soltanpur C. (2016). A Survey on Wireless Body Area Networks for Ehealthcare Systems in Residential Environments. Sensors.

[2] Yeganeh H. (2019). An Analysis of Emerging Trends and Transformations in Global Healthcare. Int. J. Health Gov..

[3] Woznowski P., Fafoutis X., Song T., Sion H., Camplani M., Lili T., Paiement A., Mellios E., Mo H., Ni Z. A Multi-Modal Sensor Infrastructure for Healthcare in a Residential Environment. Proceedings of the 2015 IEEE International Conference on Communication Workshop (ICCW).

[4] Sherratt R.S., Janko B., Hui T., Harwin W., Diaz-Sanchez D. Dictionary Memory Based Software Architecture for Distributed Bluetooth Low Energy Host Controllers Enabling High Coverage in Consumer Residential Healthcare Environments. Proceedings of the 2017 IEEE International Conference on Consumer Electronics (ICCE).

[5] Fafoutis X., Tsimbalo E., Mellios E., Hilton G., Piechocki R., Craddock I. (2016). A Residential Maintenance-Free Long-Term Activity Monitoring System for Healthcare Applications. EURASIP J. Wirel. Commun. Netw..

[6] World Health Statistics (2018). Monitoring Health for the SDGs, Sustainable Development Goals.

[7] Ludden G.D.S., Van Rompay T.J.L., Niedderer K., Tournier I. (2019). Environmental Design for Dementia Care—Towards More Meaningful Experiences through Design. Maturitas.

[8] Chen H., Xue M., Mei Z., Oetomo S.B., Chen W. (2016). A Review of Wearable Sensor Systems for Monitoring Body Movements of Neonates. Sensors.

[9] Rose K.J., Petrut C., L’heveder R., De Sabata S. (2019). Idf Europe’s Position on Mobile Applications in Diabetes. Diabetes Res. Clin. Pract..

[10] Majumder S., Aghayi E., Noferesti M., Memarzadeh-Tehran H., Mondal T., Pang Z., Deen M. (2017). Smart Homes for Elderly Healthcare-Recent Advances and Research Challenges. Sensors.

[11] Uzor S., Baillie L. (2018). Exploring the Communication of Progress in Home-Based Falls Rehabilitation Using Exergame Technologies. Proc. ACM Interact. Mob. Wearable Ubiquitous Technol..

[12] Stack E., King R., Janko B., Burnett M., Hammersley N., Agarwal V., Hannuna S., Burrows A., Ashburn A. (2016). Could in-Home Sensors Surpass Human Observation of People with Parkinson’s at High Risk of Falling? An Ethnographic Study. BioMed Res. Int..

[13] Mshali H., Lemlouma T., Moloney M., Magoni D. (2018). A Survey on Health Monitoring Systems for Health Smart Homes. Int. J. Ind. Ergon..

[14] Wang Y., Cang S., Yu H. (2019). A Survey on Wearable Sensor Modality Centred Human Activity Recognition in Health Care. Expert Syst. Appl..

[15] Buonocunto P., Giantomassi A., Marinoni M., Calvaresi D., Buttazzo G. (2018). A Limb Tracking Platform for Tele-Rehabilitation. ACM Trans. Cyber-Phys. Syst..

[16] Ni Z., Diethe T., Camplani M., Lili T., Burrows A., Twomey N., Kaleshi D., Mirmehdi M., Flach P., Craddock I. (2015). Bridging E-Health and the Internet of Things: The Sphere Project. IEEE Intell. Syst..

[17] Akther S., Saleheen N., Samiei S., Shetty V., Ertin E., Kumar S. (2019). Moral: An Mhealth Model for Inferring Oral Hygiene Behaviors in-the-Wild Using Wrist-Worn Inertial Sensors. Proc. ACM Interact. Mob. Wearable Ubiquitous Technol..

[18] Tonkin E.L., Woznowski P.R. Activities of daily living ontology for ubiquitous systems. Proceedings of the 2018 IEEE International Conference on Pervasive Computing and Communications Workshops (PerCom Workshops).

[19] Liu L., Stroulia E., Nikolaidis I., Miguel-Cruz A., Rincon A.R. (2016). Smart homes and home health monitoring technologies for older adults: A systematic review. Int. J. Med. Inform..

[20] Bennett J., Rokas O., Chen L. (2017). Healthcare in the smart home: A study of past, present and future. Sustainability.

[21] Fafoutis X., Vafeas A., Janko B., Sherratt R.S., Pope J., Elsts A., Mellios E., Hilton G., Oikonomou G., Piechocki R. (2017). Designing Wearable Sensing Platforms for Healthcare in a Residential Environment. EAI Endorsed Trans. Pervasive Health Technol..

[22] Tao L., Burghardt T., Hannuna S., Camplani M., Paiement A., Damen D., Mirmehdi M., Craddock I. A Comparative Home Activity Monitoring Study Using Visual and Inertial Sensors. Proceedings of the 17th International Conference on E-health Networking, Application & Services (HealthCom).

[23] Forkan A., Branch P., Jayaraman P., Ferretto A. (2019). An Internet-of-Things Solution to Assist Independent Living and Social Connectedness in Elderly. ACM Trans. Soc. Comput..

[24] Lee E.K., Wang Y., Davis R.A., Egan B.M. Designing a Low-Cost Adaptable and Personalized Remote Patient Monitoring System. Proceedings of the 2017 IEEE International Conference on Bioinformatics and Biomedicine (BIBM).

[25] Wang Z., Xu M., Ye N., Wang R., Huang H. (2019). Rf-Focus: Computer Vision-Assisted Region-of-Interest Rfid Tag Recognition and Localization in Multipath-Prevalent Environments. Proc. ACM Interact. Mob. Wearable Ubiquitous Technol..

[26] Fares N., Sherratt R.S., Elhajj I.H. (2021). Directing and Orienting ICT Healthcare Solutions to Address the Needs of the Aging Population. Healthcare.

[27] Chadborn N.H., Blair K., Creswick H., Hughes N., Dowthwaite L., Adenekan O., Pérez Vallejos E. (2019). Citizens’ Juries: When Older Adults Deliberate on the Benefits and Risks of Smart Health and Smart Homes. Healthcare.

[28] Baker S.B., Xiang W., Atkinson I. (2017). Internet of things for smart healthcare: Technologies, challenges, and opportunities. IEEE Access.

[29] Balta-Ozkan N., Davidson R., Bicket M., Whitmarsh L. (2013). Social barriers to the adoption of smart homes. Energy Policy.

[30] Kaziunas E., Klinkman M., Ackerman M. (2019). Precarious Interventions: Designing for Ecologies of Care. Proc. ACM Hum.-Comput. Interact..

[31] West J. (2020). Design in healthcare: The challenge of translation. Des. Health.

[32] Pedersen S. (2020). Staging negotiation spaces: A co-design framework. Des. Stud..

[33] Jones P.H. (2013). Design for Care: Innovating Healthcare Experience.

[34] Moher D., Liberati A., Tetzlaff J., Altman D.G., Prisma Group (2009). Preferred reporting items for systematic reviews and meta-analyses: The PRISMA statement. PLoS Med..

[35] Huberman M., Miles M.B. (2002). Qualitative Researcher’s Companion.

[36] Bastian M., Heymann S., Jacomy M. Gephi: An open-source software for exploring and manipulating networks. Proceedings of the Third international AAAI conference on weblogs and social media.

[37] Nthubu B. (2021). Enhancing the Understanding of Manufacturing SME Innovation Ecosystems: A Design Visualization Approach. Ph.D. Thesis.

[38] Nthubu B., Richards D., Cruickshank L. (2020). Using Visualization Models to Speculate on New Platforms for Additive Manufacturing Expansion. J. Ind. Intell. Inf..

[39] Luker K. (2008). Salsa Dancing into the Social Sciences: Research in an Age of Info-Glut.

[40] Qi J., Yang P., Min G., Amft O., Dong F., Xu L. (2017). Advanced Internet of Things for Personalized Healthcare Systems: A Survey. Pervasive Mob Comput..

[41] Gu F., Hu X., Ramezani M., Acharya D., Khoshelham K., Valaee S., Shang J. (2019). Indoor Localization Improved by Spatial Context-a Survey. ACM Comput. Surv..

[42] Caso G., De Nardis L., Di Benedetto M.G. (2015). A Mixed Approach to Similarity Metric Selection in Affinity Propagation-Based Wi-Fi Fingerprinting Indoor Positioning. Sensors.

[43] Wu J., Feng Y. Global Wi-Fi Positioning Method Based on Online Clustering Algorithm. Proceedings of the 4th International Conference on Big Data Computing and Communications (BIGCOM).

[44] Shtar G., Shapira B., Rokach L. (2019). Clustering Wi-Fi Fingerprints for Indoor–Outdoor Detection. Wirel. Netw..

[45] Chiputa M., Xiangyang L. (2018). Real-Time Wi-Fi Indoor Positioning System Based on RSSI Measurements: A Distributed Load Approach with the Fusion of Three Positioning Algorithms. Wirel. Pers. Commun..

[46] Elhamshary M., Youssef M. Semsense: Automatic Construction of Semantic Indoor Floorplans. Proceedings of the 2015 International Conference on Indoor Positioning and Indoor Navigation (IPIN).

[47] Lymberopoulos D., Liu J. (2017). The Microsoft Indoor Localization Competition: Experiences and Lessons Learned. IEEE Signal Process. Mag..

[48] Mcallister T.D., El-Tawab S., Heydari M.H. Localization of Health Center Assets through an IoT Environment (Locate). Proceedings of the 2017 Systems and Information Engineering Design Symposium (SIEDS).

[49] Bradley C., El-Tawab S., Heydari M.H. Security Analysis of an IoT System Used for Indoor Localization in Healthcare Facilities. Proceedings of the 2018 Systems and Information Engineering Design Symposium (SIEDS).

[50] Davidson P., Piche R. (2017). A Survey of Selected Indoor Positioning Methods for Smartphones. IEEE Commun. Surv. Tutor..

[51] Martinez-Sala A.S., Losilla F., Sánchez-Aarnoutse J.C., García-Haro J., Khoshelham K., Zlatanova S. (2015). Design, Implementation and Evaluation of an Indoor Navigation System for Visually Impaired People. Sensors.

[52] Wang C., Liu J., Chen Y., Xie L., Liu H., Lu S. (2018). Rf-Kinect: A Wearable Rfid-Based Approach Towards 3d Body Movement Tracking. Proc. ACM Interact. Mob. Wearable Ubiquitous Technol..

[53] Fan X., Gong W., Liu J. (2018). Tagfree Activity Identification with Rfids. Proc. ACM Interact. Mob. Wearable Ubiquitous Technol..

[54] Patel H.J., Temple M.A., Baldwin R.O. (2015). Improving Zigbee Device Network Authentication Using Ensemble Decision Tree Classifiers with Radio Frequency Distinct Native Attribute Fingerprinting. IEEE Trans. Reliab..

[55] Amiribesheli M., Benmansour A., Bouchachia A. (2015). A Review of Smart Homes in Healthcare. J. Ambient Intell. Humaniz. Comput..

[56] Yang D., Xu B., Rao K., Sheng W. (2018). Passive Infrared (PIR)-Based Indoor Position Tracking for Smart Homes Using Accessibility Maps and A-Star Algorithm. Sensors.

[57] Mossel A. (2015). Robust 3d Position Estimation in Wide and Unconstrained Indoor Environments. Sensors.

[58] Chen C., Tang L., Hancock C.M., Zhang P. (2019). Development of Low-Cost Mobile Laser Scanning for 3d Construction Indoor Mapping by Using Inertial Measurement Unit, Ultra-Wide Band and 2d Laser Scanner. Eng. Constr. Archit. Manag..

[59] Gualda D., Ureña J., García E. (2016). Partially Constrained Extended Kalman Filter for Navigation Including Mapping Information. IEEE Sens. J..

[60] Santagati G., Melodia T. (2017). A Software-Defined Ultrasonic Networking Framework for Wearable Devices. IEEE ACM Trans Netw..

[61] Hoa J., Soewito B. (2018). Monitoring Human Movement in Building Using Bluetooth Low Energy. CommIT J..

[62] Tekler Z.D., Low R., Gunay B., Andersen R.K., Blessing L. (2020). A Scalable Bluetooth Low Energy Approach to Identify Occupancy Patterns and Profiles in Office Spaces. Build Environ..

[63] Qi J., Yang P., Waraich A., Deng Z., Zhao Y., Yang Y. (2018). Examining Sensor-Based Physical Activity Recognition and Monitoring for Healthcare Using Internet of Things: A Systematic Review. J. Biomed. Inform..

[64] Zhang G., Mei Z., Zhang Y., Ma X., Lo B., Chen D., Zhang Y. (2020). A Non-Invasive Blood Glucose Monitoring System Based on Smartphone Ppg Signal Processing and Machine Learning. IEEE Trans. Ind. Inform..

[65] Kiaghadi A., Homayounfar S., Gummeson J., Andrew T., Ganesan D. (2019). Phyjama: Physiological Sensing Via Fiber-Enhanced Pyjamas. Proc. ACM Interact. Mob. Wearable Ubiquitous Technol..

[66] Hathaliya J.J., Tanwar S. (2020). An Exhaustive Survey on Security and Privacy Issues in Healthcare 4.0. Comput. Commun..

[67] Mohamed R., Youssef M. (2017). Heartsense: Ubiquitous Accurate Multi-Modal Fusion-Based Heart Rate Estimation Using Smartphones. Proc. ACM Interact. Mob. Wearable Ubiquitous Technol..

[68] Dementyev A., Hernandez J., Choi I., Follmer S., Paradiso J. (2018). Epidermal Robots: Wearable Sensors That Climb on the Skin. 2018. Proc. ACM Interact. Mob. Wearable Ubiquitous Technol..

[69] Tucker C.S., Behoora I., Nembhard H.B., Lewis M., Sterling N.W., Huang X. (2015). Machine learning classification of medication adherence in patients with movement disorders using non-wearable sensors. Comput. Biol. Med..

[70] Cunha A., Pádua L., Costa L., Trigueiros P. (2014). Evaluation of Ms Kinect for Elderly Meal Intake Monitoring. Procedia Technol..

[71] Yang Z., Pathak P., Zeng Y., Liran X., Mohapatra P. (2017). Vital Sign and Sleep Monitoring Using Millimeter Wave. ACM Trans. Sens. Netw..

[72] Liu C., Xiong J., Cai L., Feng L., Chen X., Fang D. (2019). Beyond Respiration: Contactless Sleep Sound-Activity Recognition Using Rf Signals. Proc. ACM Interact. Mob. Wearable Ubiquitous Technol..

[73] Pino E.J., Moran A.A., Dorner De La Paz A., Aqueveque P. Validation of Non-Invasive Monitoring Device to Evaluate Sleep Quality. Proceedings of the Annual International Conference of the IEEE Engineering in Medicine and Biology Society.

[74] Kamišali´ A., Fister I., Turkanovic M., Karakatic S. (2018). Sensors and Functionalities of Non-Invasive Wrist-Wearable Devices: A Review. Sensors.

[75] Whitehouse S., Yordanova K., Ludtke S., Paiement A., Mirmehdi M. Evaluation of Cupboard Door Sensors for Improving Activity Recognition in the Kitchen. Proceedings of the 2018 IEEE International Conference on Pervasive Computing and Communications Workshops (PerCom Workshops).

[76] Yuan B., Herbert J. (2014). Context-aware hybrid reasoning framework for pervasive healthcare. Pers. Ubiquitous Comput..

[77] Forkan A., Khalil I., Tari Z. (2014). CoCaMAAL: A cloud-oriented context-aware middleware in ambient assisted living. Future Gener. Comput. Syst..

[78] Pung H.K., Gu T., Xue W., Palmes P.P., Zhu J., Ng W.L., Tang C.W., Chung N.H. (2009). Context-aware middleware for pervasive elderly homecare. IEEE J. Sel. Areas Commun..

[79] Pradeep P., Krishnamoorthy S. (2019). The MOM of context-aware systems: A survey. Comput. Commun..

[80] Nugroho L.E., Lazuardi L., Non-alinsavath K. Ontology-based context aware for ubiquitous home care for elderly people. Proceedings of the 2015 2nd International Conference on Information Technology, Computer, and Electrical Engineering (ICITACEE).

[81] Aguilar J., Jerez M., Rodríguez T. (2018). CAMeOnto: Context awareness meta ontology modeling. Appl. Comput. Inform..

[82] De la Iglesia D.H., De Paz J.F., Villarrubia Gonzalez G., Barriuso A.L., Bajo J. (2018). A context-aware indoor air quality system for sudden infant death syndrome prevention. Sensors.

[83] Angsuchotmetee C., Chbeir R., Cardinale Y. (2020). MSSN-Onto: An ontology-based approach for flexible event processing in Multimedia Sensor Networks. Future Gener. Comput. Syst..

[84] Woznowski P., Burrows A., Laskowski P., Tonkin E., Craddock I. Evaluating the Use of Voice-Enabled Technologies for Ground-Truthing Activity Data. Proceedings of the 2017 IEEE International Conference on Pervasive Computing and Communications Workshops (PerCom Workshops).

[85] Chen W., Yu C., Tu C., Lyu Z., Tang J., Ou S., Fu Y., Xue Z. (2020). A Survey on Hand Pose Estimation with Wearable Sensors and Computer-Vision-Based Methods. Sensors.

[86] Holmes M., Song H., Tonkin E., Nieto M.P., Grant S., Flach P. Analysis of Patient Domestic Activity in Recovery from Hip or Knee Replacement Surgery: Modelling Wrist-Worn Wearable RSSI and Accelerometer Data in the Wild. Proceedings of the 3rd International Workshop on Knowledge Discovery in Healthcare Data.

[87] Liu F., Dabbish L., Kaufman G. (2017). Supporting Social Interactions with an Expressive Heart Rate Sharing Application. Proc. ACM Interact. Mob. Wearable Ubiquitous Technol..

[88] Al-Eidan R.M., Al-Khalifa H., Al-Salman A.M. (2018). A Review of Wrist-Worn Wearable: Sensors, Models, and Challenges. J. Sen..

[89] Beach C., Krachunov S., Pope J., Fafoutis X., Piechocki R.J., Craddock I., Casson A.J. (2018). An Ultra-Low Power Personalizable Wrist-Worn Ecg Monitor Integrated with IoT Infrastructure. IEEE Access.

[90] Krachunov S., Beach C., Casson A.J., Pope J., Fafoutis X., Piechocki R.J., Craddock I. Energy Efficient Heart Rate Sensing Using a Painted Electrode Ecg Wearable. Proceedings of the 2017 Global Internet of Things Summit (GIoTS).

[91] Brena R.F., García-Vázquez J.P., Galván-Tejada C.E., Muñoz-Rodriguez D., Vargas-Rosales C., Fangmeyer J. (2017). Evolution of Indoor Positioning Technologies: A Survey. J. Sen..

[92] Hall J., Hannuna S., Camplani M., Mirmehdi M., Damen D., Burghardt T., Tao L., Paiement A., Craddock I. Designing a Video Monitoring System for Aal Applications: The Sphere Case Study. Proceedings of the 2nd IET International Conference on Technologies for Active and Assisted Living (TechAAL 2016).

[93] Li X., Eckert M., Martinez J.F., Rubio G. (2015). Context aware middleware architectures: Survey and challenges. Sensors.

[94] Sykes E.R., Pentland S., Nardi S. (2015). Context-aware mobile apps using iBeacons: Towards smarter interactions. CASCON.

[95] Motti V.G. (2020). Wearable Interaction.

[96] Lou Z., Wang L., Jiang K., Wei Z., Shen G. (2020). Reviews of Wearable Healthcare Systems: Materials, Devices and System Integration. Mater. Sci. Eng. R..

[97] Roberts C., Mort M. (2009). Reshaping What Counts as Care: Older People, Work and New Technologies. Alter—Eur. J. Disabil. Res..

[98] Harold T. (2013). Technology and the Future of Healthcare. J. Public Health Res..

[99] Percival J., Hanson J. (2006). Big Brother or Brave New World? Telecare and Its Implications for Older People’s Independence and Social Inclusion. Crit. Soc. Policy.

[100] Burrows A., Gooberman-Hill R., Coyle D. Shared Language and the Design of Home Healthcare Technology. Proceedings of the 34th Annual ACM Conference on Human Factors in Computing Systems (CHI’16).

[101] Burrows A., Coyle D., Gooberman-Hill R. (2018). Privacy, Boundaries and Smart Homes for Health: An Ethnographic Study. Health Place.

[102] Lazar J., Woglom C., Chung J., Schwartz A., Grace Hsieh Y., Moore R., Crowley D., Skotko B. (2018). Co-Design Process of a Smart Phone App to Help People with Down Syndrome Manage Their Nutritional Habits. J. Usability Stud..

[103] Aceto G., Persico V., Pescapé A. (2020). Industry 4.0 and Health: Internet of Things, Big Data, and Cloud Computing for Healthcare 4.0. J. Ind. Inf. Integr..

[104] Hawley-Hague H., Boulton E., Hall A., Pfeiffer K., Todd C. (2014). Older Adults’ Perceptions of Technologies Aimed at Falls Prevention, Detection or Monitoring: A Systematic Review. Int. J. Med. Inform..

[105] Bajones M., Fischinger D., Weiss A., Puente P., Wolf D., Vincze M., Körtner T., Weninger M., Papoutsakis K., Michel D. (2019). Results of Field Trials with a Mobile Service Robot for Older Adults in 16 Private Households. ACM Trans. Hum.-Robot Interact..

[106] Pirzada P., White N., Wilde A. Sensors in Smart Homes for Independent Living of the Elderly. Proceedings of the 2018 5th International Multi-Topic ICT Conference.

[107] Fenza G., Furno D., Loia V. (2012). Hybrid Approach for Context-Aware Service Discovery in Healthcare Domain. J. Comput. Syst. Sci..

[108] Dhanvijay M.M., Patil S.C. (2019). Internet of Things: A Survey of Enabling Technologies in Healthcare and Its Applications. Comput. Netw..

[109] Ni Q., García Hernando A.B., Pau de la Cruz I. (2016). A context-aware system infrastructure for monitoring activities of daily living in smart home. J. Sens..

[110] Mileo A., Merico D., Bisiani R. (2010). Support for context-aware monitoring in home healthcare. J. Ambient Intell. Smart Environ..

[111] Nava-Muñoz S., Morán A.L. (2012). CANoE: A context-aware notification model to support the care of older adults in a nursing home. Sensors.

[112] Alkhomsan M.N., Hossain M.A., Rahman S.M.M., Masud M. (2017). Situation awareness in ambient assisted living for smart healthcare. IEEE Access.

[113] Alirezaie M., Renoux J., Köckemann U., Kristoffersson A., Karlsson L., Blomqvist E., Tsiftes N., Voigt T., Loutfi A. (2017). An ontology-based context-aware system for smart homes: E-care@ home. Sensors.

[114] Wan L., Müller C., Randall D., Wulf V. (2016). Design of a Gps Monitoring System for Dementia Care and Its Challenges in Academia-Industry Project. ACM Trans. Comput.-Hum. Interact. (TOCHI).

[115] Soto-Mendoza V., García-Macías J.A., Chavez E., Martínez-García A.I., Favela J., Serrano-Alvarado P., Rojas M.R.Z. (2015). Design of a predictive scheduling system to improve assisted living services for elders. ACM Trans. Intell. Syst. Technol..

[116] Hsu C.-C., Lin B.S., He K.Y., Lin B.S. (2019). Design of a Wearable 12-Lead Noncontact Electrocardiogram Monitoring System. Sensors.

[117] Anchana M., Mahasak K. (2019). The Real-Time Electrocardiogram Signal Monitoring System in Wireless Sensor Network. Int. J. Online Biomed. Eng..

[118] Seol T., Lee S., Lee J. A Wearable Electrocardiogram Monitoring System Robust to Motion Artifacts. Proceedings of the 2018 International SoC Design Conference.

[119] Burrows A., Gooberman-Hill R., Coyle D. Home Truths: Insights for Designing Inclusive Smart Home Technologies for Healthcare. Proceedings of the Design4Health Proceedings of the Third European Conference on Design4Health.

[120] Pereyda C., Raghunath N., Minor B., Wilson G., Schmitter-Edgecombe M., Cook D. (2019). Cyber-Physical Support of Daily Activities: A Robot/Smart Home Partnership. ACM Trans. Cyber-Phys. Syst..

[121] Chen Y., Abel K.T., Janecek J.T., Chen Y., Zheng K., Cramer S.C. (2019). Home-Based Technologies for Stroke Rehabilitation: A Systematic Review. Int. J. Med. Inform..

[122] Kim Y., Jung H.T., Park J., Kim Y., Ramasarma N., Bonato P., Choe E., Lee S. (2019). Towards the Design of a Ring Sensor-Based Mhealth System to Achieve Optimal Motor Function in Stroke Survivors. Proc. ACM Interact. Mob. Wearable Ubiquitous Technol..

[123] Giles B., Richard H., Madeleine M., Ruud Ter M., Peter F., Rachael G.H. (2017). Smart Homes, Private Homes? An Empirical Study of Technology Researchers’ Perceptions of Ethical Issues in Developing Smart-Home Health Technologies. BMC Med. Ethics.

[124] Chan M., Estève D., Fourniols J.Y., Escriba C., Campo E. (2012). Smart Wearable Systems: Current Status and Future Challenges. Artif. Intell. Med..

[125] Cozza M., De Angeli A., Tonolli L. (2017). Ubiquitous Technologies for Older People. Pers. Ubiquitous Comput..

[126] Washington A.L., Kuo R. (2020). Whose Side Are Ethics Codes On? Power, Responsibility and the Social Good. Proceedings of the 2020 Conference on Fairness, Accountability, and Transparency (FAT* ’20), Barcelona, Spain, 27–30 January 2020.

[127] Gandhi V., Singh J. (2020). An Automated Review of Body Sensor Networks Research Patterns and Trends. An Automated Review of Body Sensor Networks Research Patterns and Trends. J. Ind. Inf. Integr..

[128] Durcinoska I., Chung K., Young J., Solomon M. Social Networks and Healthcare Coordination: Lessons Learned from an Australian Cancer Care Survey. Proceedings of the 2017 IEEE/ACM International Conference on Advances in Social Networks Analysis and Mining 2017.

[129] Sholla S., Naaz R., Chishti M.A. Incorporating Ethics in Internet of Things (Iot) Enabled Connected Smart Healthcare. Proceedings of the 2017 IEEE/ACM International Conference on Connected Health: Applications, Systems and Engineering Technologies.

[130] Winfield A.F., Michael K., Pitt J., Evers V. (2019). Machine ethics: The design and governance of ethical AI and autonomous systems [scanning the issue]. Proc. IEEE.

[131] Sandlund M., Skelton D.A., Pohl P., Ahlgren C., Melander-Wikman A., Lundin-Olsson L. (2017). Gender Perspectives on Views and Preferences of Older People on Exercise to Prevent Falls: A Systematic Mixed Studies Review. BMC Geriatr..

[132] Burrows A., Gooberman-Hill R., Coyle D. (2015). Empirically Derived User Attributes for the Design of Home Healthcare Technologies. Pers Ubiquit Comput.

[133] Stankovic J. (2017). Research Directions for Cyber Physical Systems in Wireless and Mobile Healthcare. ACM Trans. Cyber-Phys. Syst..

[134] Tang W., Ren J., Zhang K., Zhang D., Zhang Y., Shen X. (2019). Efficient and Privacy-Preserving Fog-Assisted Health Data Sharing Scheme. ACM Trans. Intell. Syst. Technol..

[135] Parvin P., Chessa S., Manca M., Paterno F. (2018). Real-Time Anomaly Detection in Elderly Behavior with the Support of Task Models. Proc. ACM Hum. Comput. Interact..

[136] Alami A., Benhlima L., Bah S. A study of security requirements in wireless sensor networks for smart home healthcare systems. Proceedings of the 3rd International Conference on Smart City Applications.

[137] Yuehong Y.I.N., Zeng Y., Chen X., Fan Y. (2016). The internet of things in healthcare: An overview. J. Ind. Inf. Integr..

[138] Pham M., Mengistu Y., Do H.M., Sheng W. Cloud-based smart home environment (CoSHE) for home healthcare. Proceedings of the 2016 IEEE International Conference on Automation Science and Engineering.

[139] Khaloufi H., Abouelmehdi K., Beni-Hssane A. Fog Computing for Smart Healthcare data Analytics: An Urgent Necessity. Proceedings of the 3rd International Conference on Networking, Information Systems & Security.

[140] Al Muhtadi J., Alamri R.A., Khan F.A., Saleem K. (2021). Subjective logic-based trust model for fog computing. Comput. Commun..

[141] Tang W., Zhang K., Zhang D., Ren J., Zhang Y., Shen X.S. (2019). Fog-Enabled Smart Health: Toward Cooperative and Secure Healthcare Service Provision. IEEE Commun. Mag..

[142] Al Hamid H.A., Rahman S.M.M.R., Hossain M.S., Almogren A., Alamari A. (2017). A Security Model for Preserving the Privacy of Medical Big Data in a Healthcare Cloud Using a Fog Computing Facility with Pairing-Based Cryptography. IEEE Access.

[143] Rahimi M., Songhorabadi M., Kashani M.H. (2020). Fog-based smart homes: A systematic review. J. Netw. Comput..

[144] Maher N.A., Senders J.T., Hulsbergen A.F.C., Lamba N., Parker M., Onnela J.P., Bredenoord A.L., Smith T.R., Broekman M.L.D. (2019). Passive Data Collection and Use in Healthcare: A Systematic Review of Ethical Issues. Int. J. Med. Inform..

[145] Bhatia M., Sood S.K. (2017). A Comprehensive Health Assessment Framework to Facilitate Iot-Assisted Smart Workouts: A Predictive Healthcare Perspective. Comput. Ind..

[146] Kristina Y., Stefan L., Samuel W., Frank K., Adeline P., Majid M., Ian C., Thomas K. (2019). Analysing Cooking Behaviour in Home Settings: Towards Health Monitoring. Sensors.

[147] Fang L., Yin C., Zhu J., Ge C., Tanveer M., Jolfaei A., Cao Z. (2020). Privacy protection for medical data sharing in smart healthcare. ACM Trans. Multimed. Comput. Commun. Appl..

[148] Ganti R., Pham N., Tsai Y.E., Abdelzaher T. Poolview: Stream Privacy for Grassroots Participatory Sensing. Proceedings of the 6th ACM conference on Embedded network sensor systems (SenSys ’08).

[149] Theodouli A., Arakliotis S., Moschou K., Votis K., Tzovaras D. On the Design of a Blockchain-Based System to Facilitate Healthcare Data Sharing. Proceedings of the 2018 17th IEEE International Conference on Trust, Security And Privacy In Computing And Communications/ 12th IEEE International Conference On Big Data Science And Engineering (TrustCom/BigDataSE).

[150] McGhin T., Choo K.K.R., Liu C.Z., He D. (2019). Blockchain in healthcare applications: Research challenges and opportunities. J. Netw. Comput. Appl..

[151] Ahi A., Singh A.V. Role of Distributed Ledger Technology (Dlt) to Enhance Resiliency in Internet of Things (Iot) Ecosystem. Proceedings of the 2019 Amity International Conference on Artificial Intelligence.

[152] Islam S.M.R., Kwak D., Kabir M.H., Hossain H., Kwak K. (2015). The Internet of Things for Health Care: A Comprehensive Survey. IEEE Access.

[153] Escoffery C., Mcgee R., Bidwell J., Sims C., Thropp E.K., Frazier C., Mynatt E.D. (2018). A Review of Mobile Apps for Epilepsy Self-Management. Epilepsy Behav..

[154] Shegog R., Braverman L., Hixson J.D. (2020). Digital and Technological Opportunities in Epilepsy: Toward a Digital Ecosystem for Enhanced Epilepsy Management. Epilepsy Behav..

[155] Park Y., Ho J.C. Califorest: Calibrated Random Forest for Health Data. Proceedings of the ACM Conference on Health, Inference, and Learning (CHIL ’20).

[156] Batalla J.M., Vasilakos A., Gajewski M. (2017). Secure smart homes: Opportunities and challenges. ACM Comput. Surv..

[157] Fang R., Pouyanfar S., Yang Y., Chen S.C., Iyengar S.S. (2016). Computational Health Informatics in the Big Data Age: A Survey. ACM Comput. Surv..

[158] Nakarada-Kordic I., Kayes N., Reay S., Wrapson J., Collier G. (2020). Co-creating health: Navigating a design for health collaboration. Des. Health.

[159] Rowe A., Knox M., Harvey G. (2020). Re-thinking health through design: Collaborations in research, education and practice. Des. Health.

[160] Groeneveld B., Dekkers T., Boon B., D’Olivo P. (2018). Challenges for design researchers in healthcare. Des. Health.

[161] Foucault M. (1973). The Birth of the Clinic: An Archaeology of Medical Perception.

[162] Chamberlain P., Partridge R. (2017). Co-designing co-design. Shifting the culture of practice in healthcare. Des. J..

[163] Akoglu C., Dankl K. (2019). Co-creation for empathy and mutual learning: A framework for design in health and social care. CoDesign.

[164] Spinelli G., Micocci M., Martin W., Wang Y. (2019). From medical devices to everyday products: Exploring cross-cultural perceptions of assistive technology. Des. Health.

[165] Sarantou M., Pan S. (2020). Design for society: Ageing communities as co-designers in processes of social innovation. J. Des. Bus. Soc..

[166] Veldmeijer L., Wartena B., Terlouw G., van’t Veer J. (2020). Reframing loneliness through the design of a virtual reality reminiscence artefact for older adults. Des. Health.

[167] Huang T.T.K., Aitken J., Ferris E., Cohen N. (2018). Design thinking to improve implementation of public health interventions: An exploratory case study on enhancing park use. Des. Health.

[168] Zanutto A. (2019). ‘Two clicks and I’m in!’Patients as co-actors in managing health data through a personal health record infrastructure. Health Inform. J..

[169] Yassine A., Singh S., Hossain M.S., Muhammad G. (2019). IoT big data analytics for smart homes with fog and cloud computing. Future Gener. Comput. Syst..

